# Uptake of Trastuzumab Biosimilars for the Treatment of HER2-Positive Breast Cancer: A Real-World Experience from a Cancer Center

**DOI:** 10.3390/pharmaceutics13050684

**Published:** 2021-05-10

**Authors:** Michela Piezzo, Roberta D’Aniello, Ilaria Avallone, Bruno Barba, Daniela Cianniello, Stefania Cocco, Antonio D’Avino, Germira Di Gioia, Vincenzo Di Lauro, Giuseppina Fusco, Raffaele Piscitelli, Claudia von Arx, Michelino De Laurentiis, Piera Maiolino

**Affiliations:** 1 Department of Breast and Thoracic Oncology, Division of Breast Medical Oncology, Istituto Nazionale Tumori IRCCS “Fondazione G. Pascale”, 80131 Naples, Italy; d.cianniello@istitutotumori.na.it (D.C.); s.cocco@istitutotumori.na.it (S.C.); g.digioia@istitutotumori.na.it (G.D.G.); v.dilauro@istitutotumori.na.it (V.D.L.); g.fusco@istitutotumori.na.it (G.F.); claudia.vonarx@istitutotumori.na.it (C.v.A.); 2 Pharmacy, Istituto Nazionale Tumori IRCCS “Fondazione G. Pascale”, 80131 Naples, Italy; r.daniello@istitutotumori.na.it (R.D.); b.barba@istitutotumori.na.it (B.B.); a.davino@istitutotumori.na.it (A.D.); rpiscitelli8@gmail.com (R.P.); p.maiolino@istitutotumori.na.it (P.M.); 3 Department of Experimental Medicine, Azienda Ospedaliera Universitaria degli Studi della Campania “Luigi Vanvitelli”, 80138 Naples, Italy; ilaria_avallone@libero.it

**Keywords:** breast cancer, trastuzumab, biosimilars, HER2

## Abstract

Background: The introduction of trastuzumab biosimilars in clinical practice plays an important role in promoting the sustainability of healthcare systems. By contrast, the switching process can be challenging to the clinics. This survey describes the switching process at a National Cancer Institute over a period of 2 years. Methods: Data regarding all trastuzumab-based regimens for breast cancer (BC) from 1 January 2019 and 31 December 2020 were extracted from both adverse drug reactions (ADRs) reporting systems and electronic systems involved in inventory management, prescribing, dispensing, and administration. Both patients under monotherapy and combination treatment regimens were included. There were no exclusion criteria. Results and Conclusions: Overall 354 patients received at least one trastuzumab-based regimen for a total of 493 lines of treatment and 5769 administrations. Biosimilar were used in 34.3% of trastuzumab-based treatments. No differences between biosimilars and reference drug have been observed in terms of ADRs. The effective cost-saving of the first 2 years is greater than EUR 800,000 and it is estimated to increase over time.

## 1. Introduction

Overexpression of human epidermal growth factor receptor-2 (HER2) is observed in 20–30% of human breast cancers and is associated with a more aggressive phenotype, higher rates of recurrence, and increased mortality compared with HER2-negative BC [1,2]. The introduction of HER2-directed therapies markedly improved the outcome of these patients [3,4]. Trastuzumab, a recombinant humanized monoclonal antibody that binds to the extracellular subdomain IV of HER2, is the first anti-HER2 agent approved for the treatment of HER2-positive metastatic and early BC [5,6,7]. Trastuzumab is routinely continued over a period of 1 year as adjuvant treatment for early HER2-positive BC [8], while it is continued until and beyond disease progression as treatment for metastatic HER2-positive BC. Therefore, re-therapy with trastuzumab has been demonstrated to be effective and safe [9,10,11,12]. For long treatment periods, several efforts have been made to reduce the application time and optimize the application route, leading to the development of a subcutaneous (SC) formulation of trastuzumab, first approved in Europe in 2013 and subsequently in the U.S. in 2019. The SC formulation, administered every 3 weeks (Q3W) as a fixed dose of 600 mg in a 5 mL volume, allows a fast and simple administration within 2–5 min without the need for venous access [13,14]. Although trastuzumab-based therapy is the standard of care among these patients, observational studies on real-world treatment patterns suggest that 12% to 54% of patients do not receive trastuzumab or any other anti-HER2 agent due to drug funding and high treatment costs, resulting in disparities in the access to therapy for many patients, especially in low-middle income countries, implying significant survival gaps in the world [9,15].

The trastuzumab European patent expired in 2014 and the US patent expired in 2019, leading several producers to submit applications for trastuzumab biosimilars, allowing, in the future, for a greater use of anti-HER2 agents. In 2019, the World Health Organization (WHO) included the biosimilars of trastuzumab in the WHO essential list of medicines (EML), recognizing the value of biosimilars as therapeutically equivalent alternatives able to improve patient access and cost-effective use of resources by a greater market competition [16,17]. Similar to the Food and Drug Administration (FDA), the European Medicine Agency (EMA) defines biosimilars as “products that contain a version of the active substance of an already authorized original biological medicinal product,” being similar “in terms of quality characteristics, biological activity, safety and efficacy based on a comprehensive comparability exercise” [18]. According to this definition, biosimilars can induce the same clinical outcome in a single patient, but they are produced in different cell lines or bacteria, starting from a DNA sequence which encodes a protein subjected to various post-translational changes. These unknown changes in folding or posttranslational structure can raise potential concerns related to structural variability and immunogenicity. The market authorization for biosimilars is based on a stepwise approach, also known as “Totally of Evidence”, aimed to demonstrate identical biological characteristics, efficacy, and safety to the reference drug. Once the similarity has been demonstrated at the molecular level, the preclinical and clinical testing may be shorter than the marketing authorization process for new drugs. The phase I studies must demonstrate the equivalence in terms of pharmacodynamics and pharmacokinetics, and phase III studies must provide evidence in terms of safety and efficacy in at least one indication. Once this evidence has been provided, the marketing authorization may be granted in the indication studied, as well as in all additional indications, of the reference drug if the mechanism of action of biosimilar is the same in all indications [19,20,21,22,23]. 

To date, five trastuzumab biosimilars have already been authorized in Italy for the treatment of patients with HER2-positive early or metastatic BC: Kanjinti^®^ (Amgen, Thousand Oaks, CA, USA), Ontruzant^®^ (MSD), Herzuma^®^ (Mundipharma, Hong Kong, China), Ogivri^®^ (Mylan, Canonsburg, PA, USA), and Trazimera^®^ (Pfizer, New York, NY, USA) [24,25,26,27]. Clinical efficacy and safety of trastuzumab biosimilars have been rigorously tested through the comparability exercise in moderately sized, randomized, double-blind phase III studies in neoadjuvant or metastatic settings [28,29,30,31,32]. Nonetheless, they still require continuous monitoring once the biosimilar is approved [33,34]. The results have shown no evidence of potential clinical safety concerns associated with biosimilars administration, no toxicity findings in animals, and similar PK profiles in humans [35]. However, there is some evidence of the preclinical and clinical combination of biosimilars and chemotherapy drugs or other biological therapies, such as pertuzumab, a humanized monoclonal antibody that interacts with a different HER2 epitope than trastuzumab, resulting in a clinical advantage and greater activity than trastuzumab alone [36,37,38,39,40]. For these reasons, both physicians and patients still have concerns regarding the efficacy, safety, and exchangeability of these drugs. 

The survey presented here summarizes the organizational and clinical experience from the National Cancer Institute of Naples during the switching process from the reference trastuzumab to biosimilars. 

## 2. Materials and Methods

This was an observational, population-based survey recording the switch from originator to biosimilar of targeted anti-HER2 therapy for early and metastatic, HER2-positive breast cancer at the National Cancer Institute of Naples. The objective of this survey was to follow the switching process from trastuzumab originator to biosimilars in terms of safety and cost-saving over a period of 2 years. Access to trastuzumab biosimilars was granted at our institution from January 2019. Data regarding all trastuzumab-based regimens for BC from 1 January 2019 and 31 December 2020 were extracted from both adverse drug reactions (ADRs) reporting systems and electronic systems involved in inventory management, prescribing, dispensing, and administration. Both patients under monotherapy and combination treatment regimens were included. There were no exclusion criteria. The starting dates of each biosimilar are not the same because the hospital adopted some biosimilars earlier than others. The switch between the reference trastuzumab and its biosimilars, or among different biosimilars, was managed at the discretion of the treating physician on a case-by-case basis in partnership with the patient, considering the pharmacy availability. 

As per clinical practice, all suspected ADRs were recorded and monitored by clinicians and pharmacists involved in pharmacovigilance activity. Since the ADR case report can refer to more than one ADR for a single patient in association with a single administration, a standardized procedure was implemented to handle data contained in ADR case reports. Each ADR was coded using the Medical Dictionary for Regulatory Activities (MedDRA) [41]. 

Cost assessment was performed considering the exact number of doses administered; a loading and maintenance dose of 8 mg/kg and 6 mg/kg, respectively (with exception of SC formulation, administered at fixed dose of 600 mg); a mean weight of 70 kg for dose calculation; and market prices charged to the Hospital, including value added tax (VAT). Then, for each patient, the cost of the expected standard treatment (reference trastuzumab) that shuld be administered in the absence of biosimilar drugs was also calculated to quantify the cost-saving over the observed period.

Descriptive analysis was performed using R 3.4.1 software packages (released June 2017, R Foundation for Statistical Computing, Vienna, Austria).

## 3. Results

Until 2018, before the introduction of trastuzumab biosimilars to the Italian market, trastuzumab-based regimens were regularly provided for neoadjuvant, adjuvant, and metastatic treatment of patients with HER2-positive BC, both as intravenous (IV) and subcutaneous (SC) formulation. SC administration was widely used in the adjuvant setting, after the completion of chemotherapy, and in the metastatic setting, both as monotherapy and in association with oral chemotherapy. 

At the end of 2018, the switching process was widely discussed via personal discussions and team meetings involving the pharmacists, physicians, and management department. All available trastuzumab biosimilars were discussed, and three of them were introduced in clinical practice. The ordering system was updated so that the name of the biosimilar was clearly distinguishable from the reference drug and traceability was guaranteed. Physicians initially decided not to pursue the strategy of *non-medical-switch* and to be cautious in switching stable patients [42], taking any decision to switch on a case-by-case basis together with the patient, choosing the appropriate treatment for both naïve and pretreated patients. Based on this approach, in 2019, the biosimilar was only used in newly initiated IV trastuzumab therapies with exception of pertuzumab and trastuzumab combination therapies, while the ongoing therapies (IV and SC) were continued with the reference drug (originator).

From 1 January 2019 to 31 December 2020, only 354 patients received at least one trastuzumab-based regimen, for a total of 493 lines of treatment and 5769 administrations. The characteristics of treatments are shown in Table 1. Biosimilars were used in 34.3% of trastuzumab-based treatments, mainly in the adjuvant setting and in combination with chemotherapy, while only 5.5% of biosimilar treatments were used as maintenance monotherapy. Kanjinti, the first biosimilar introduced in our clinical practice, is primarily used in the neo/adjuvant setting, while Herzuma and Ontruzant are primarily used in the recurrent/metastatic setting. Looking at the utilization of biosimilars overall, their use has gradually increased over time, with a total of 486 cycles over 2650 IV administrations in 2019 and 941 cycles over 3119 in 2020 (18.3% in 2019 vs. 30.2% in 2020).

**Figure 1:**
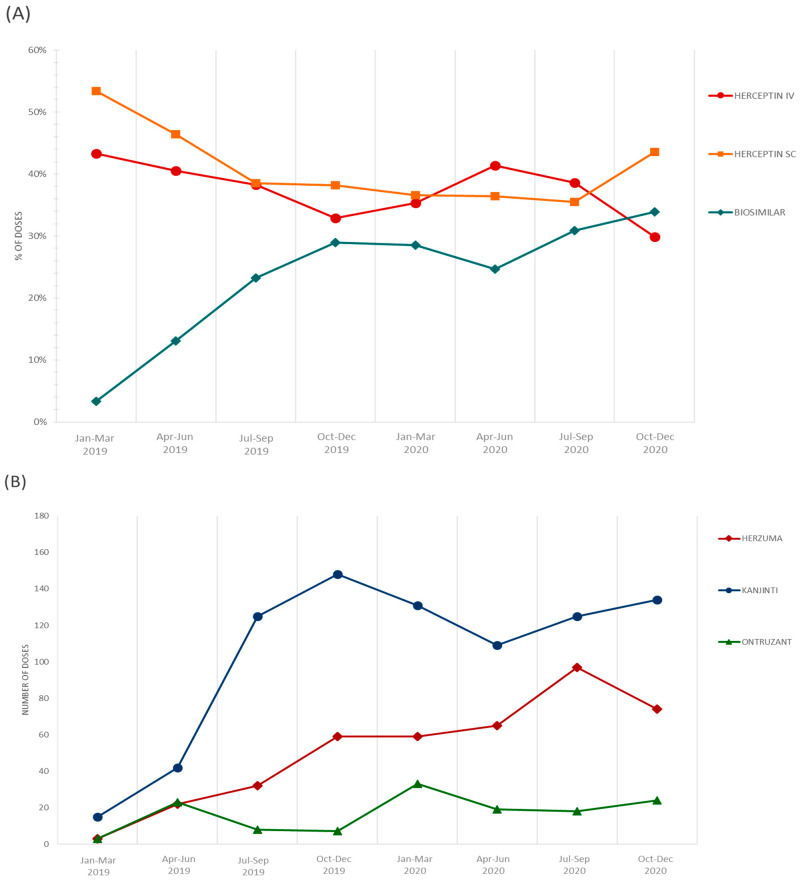
Quarterly total use of trastuzumab: (**A**) Percentage of doses of trastuzumab; (**B**) absolute number of doses divided by biosimilar.

Excluding the SC formulation of Herceptin, which is more useful for a large number of patients, the impact of introduction of biosimilars seems more impressive, with 486 cycles over a total of 1507 IV administrations in 2019 and 941 cycles over 1955 in 2020 (32.2% in 2019 vs. 48.1% in 2020). Figure 1, panel A, shows how the overall use of biosimilars has increased over time, exceeding the threshold of 30% from the third quarter of 2020. By contrast, the use of reference trastuzuamb (Herceptin IV) has slowly decreased with exception of the second quarter of 2020, during which there were some issues with the supply of Kanjinti, the most frequently used among biosimilar (panel B). The use of trastuzumab SC was quite stable over time, representing about 40% of the total doses administered. Due to its route of administration, this rate seems destined to grow in the future.

The switching process from the reference drug to biosimilar was adopted very gradually. During the first months, the majority of switches concerned the biosimilar versus Herceptin SC, especially in the adjuvant setting. After the first “adjustment period,” physicians decided to switch patients in the metastatic setting after the progression of disease. Then, at the end of 2020, the use of biosimilars was also introduced in the subgroup of patients treated with the combination of trastuzumab plus pertuzumab as first-line therapy. Table 2 shows the characteristics of switches from reference trastuzumab (originator) versus biosimilar and vice versa. The switch occurred in 22.3% of treatments (110 switches over a total of 493 treatments), of which the most frequent were the switch from biosimilar versus SC formulation (50.0%) or from originator (IV and SC) versus biosimilar (31.1%). Only 34 treatments over a total of 493 were switched from originator to biosimilar, which is consistent with the intention of using biosimilars in trastuzumab-naïve patients and from the beginning of a new line of therapy for pretreated metastatic patients.

No substantial differences were observed in the safety profile of biosimilars, and the observed ADRs of both reference trastuzumab (IV and SC) and biosimilars did not differ from those reported in the Summary of Product Characteristics, as shown in Table 3. General and gastrointestinal disorders were described for all drugs, as well as blood and lymphatic system disorders and cardiac disorders. Overall, 83 suspected ADRs were registered, of which 49 (59.0%) occurred in the metastatic setting and 22 were classified as serious. All ARDs were completely recovered/resolved at the time of data analysis.

**Figure 2:**
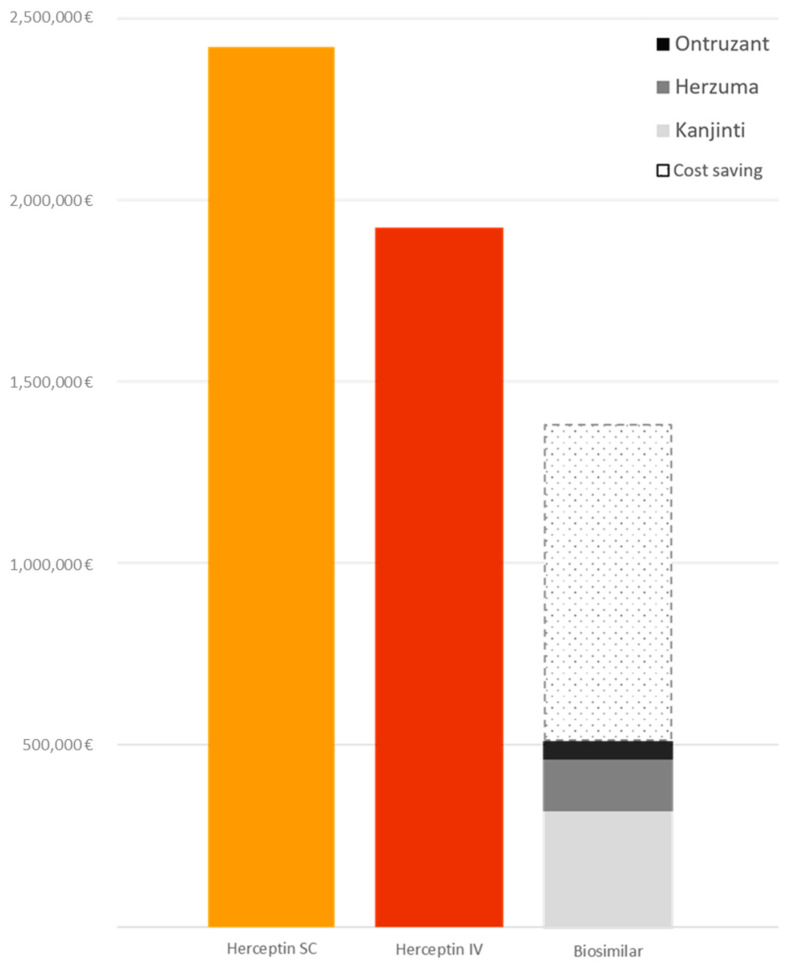
Cost assessment of the total use of trastuzumab.

Cost assessment was performed as previously described, considering the overall period of 2 years (Figure 2). The 2-year actual cost of trastuzumab amounted to EUR 4674.502, of which EUR 2171.867 refer to SC formulation of the reference drug, EUR 1992.286 refer to IV formulation of the reference drug, and EUR 510.348 refer to biosimilars. The effective cost-saving was EUR 866.678, calculated as if all patients received only the reference drug. The reference trastuzumab IV was still widely used, since the 47% of patients (232) were treated in the metastatic setting and many of them represent the long-term trastuzumab-treated patients. As previously reported, we preferred not to change type of trastuzumab in stable patients among a single line of therapy, with the exception of the switch to SC formulation.

## 4. Discussion

The introduction of trastuzumab, initially approved in 1998 by the U.S. Food and Drug Administration (FDA), deeply changed the trajectory and outcome of HER2-positive BC patients. Today, trastuzumab is the standard of care in both early and advanced settings. As with the majority of biologic drugs, trastuzumab is relatively expensive compared with traditional chemotherapy, often affecting the health care budget. The approval of trastuzumab biosimilars as therapeutically equivalent alternatives can contribute to the sustainability of cancer care by reducing costs for health care system and improving patient access worldwide. Nevertheless, the introduction of biosimilars into clinical practice can present challenges for both treating physicians and patients regarding the exchangeability of these biological drugs in terms of safety and efficacy. 

This survey collected data from a cohort of BC patients treated with trastuzumab (originator or biosimilar) in a real-life setting. Data were extracted from both ADRs reporting systems and electronic systems involved in inventory management, prescribing, dispensing, and administration. Trastuzumab biosimilars were first introduced at our National Cancer Center in 2019, showing a slow but steady increase in the utilization of these drugs. During the first year, patients treated with the combination of trastuzumab and pertuzumab, as well as long-term stable patients, were excluded from the switching process. The driving principle was to balance the level of evidence against the level of uncertainty case by case, excluding the practice of nonmedical switching for economic or other nonmedical reasons. 

Biosimilars were mainly used in trastuzumab-naïve patients and at the beginning of a new line of therapy for pretreated metastatic patients. The majority of patients treated with biosimilars in the neoadjuvant therapy were switched to SC formulation of the reference drug after surgery. Since the SC formulation was well tolerated and preferred by patients, its use has increased consistently over time.

The results of pharmacovigilance activity did not show any significant difference in terms of safety between biosimilars and reference trastuzumab, which is consistent with conclusion from health authority regulators in Europe, previous real-world studies, and a meta-analysis of randomized controlled trials [43,44,45,46]. Our results indicate that ADRs were reported in 16.8% of treatments, more frequently associated with metastatic setting and with combination therapy of trastuzumab plus chemotherapy (77% of all ADRs). Herceptin SC was the most well-tolerated trastuzumab, since it was mainly administered in the adjuvant setting or as maintenance monotherapy in the metastatic setting (Table 3). Due to differences in the number of treatments for each drug, a more detailed comparison of safety profile is not appropriate, and it is beyond the scope of this survey.

Cost analysis shows that the average annual expense for trastuzumab-based treatments exceeded the quota of EUR 2,000,000, half of which was allocated to SC formulation, with an average annual cost-saving of about EUR 400,000. The use of SC formulation, available only for the reference drug, is destined to increase over time since it offers several advantages in terms of timing of administration and less commitment by supportive health professionals, and it also requires less availability of appropriate clinical spaces. Its use is suitable for patients treated with trastuzumab alone or in combination with oral drugs (chemotherapy or endocrine therapies), while the IV formulation is more suitable for patients treated with combination of trastuzumab (+/− pertuzumab) and IV chemotherapy. This is the proportion of patients that would be switched to biosimilars in the next months to increase the effective cost-saving.

## 5. Conclusions

Biosimilars, if appropriately used, play an important role in promoting the sustainability of health care systems and allowing financial headroom for investments in new technologies and therapies. By contrast, the complex nature of biological molecules requires a balance between the level of evidence and the level of risk in each case. The approach of “one size fits all” seems not to be appropriate in this context, and the promotion of suitable clinical use of biosimilars comes through the cooperation of all parties involved, particularly physicians, pharmacists, the management department, and patients. The present survey was aimed to describe the switching process from reference trastuzumab to its biosimilars. This process presented several challenges due to concerns about the exchangeability of these drugs. Biosimilars were introduced following a stepwise process based on few simple criteria such as the line of treatment and the decision to guarantee the therapeutic continuity and not to pursue the strategy of *nonmedical switch.* The unavailability of all biosimilars over time did not allow us to compare efficacy of reference trastuzumab versus biosimilars, and this will be evaluated in the future. Overall, our experience highlights the need to implement a standardized procedure for expected future switching processes, since the availability of biosimilar drugs may lead to substantial cost-saving for healthcare system.

## Figures and Tables

**Table 1:** Characteristics of patients and treatments.

	Herceptin IV (*n*, %)	Herceptin SC (*n*, %)	Herzuma (*n*, %)	Kanjinti (*n*, %)	Ontruzant (*n*, %)
Lines of treatment	144 (29.2%)	180 (36.5%)	53 (10.8%)	100 (20.3%)	16 (3.2%)
Cycles	2035 (35.3%)	2307 (40.0%)	445 (7.7%)	844 (14.6%)	138 (2.4%)
Age of patients, year					
Median	54.6	56.9	56.4	56.7	61.6
Range	31.3–84.6	32.9–89.4	36.0–83.4	32.0–87.2	34.7–88.7
Setting					
Neoadjuvant (*n*, %)	15 (10.4%)	5 (2.8%)	4 (7.5%)	11 (11.0%)	3 (18.8%)
Adjuvant (*n*, %)	36 (25.0%)	124 (68.9%)	10 (18.9%)	50 (50.0%)	3 (18.8%)
Recurrent/Metastatic (*n*, %)	93 (64.6%)	51 (28.3%)	39 (73.6%)	39 (39.9%)	10 (62.5%)
Regimen					
T (*n*, %)	3 (2.1%)	169 (93.9%)	5 (0.9%)	4 (4.0%)	1 (0.6%)
T + P (*n*, %)	44 (30.6%)	1 (0.06%)	4 (0.7%)	9 (9.0%)	3 (18.8%)
T + CT (*n*, %)	29 (20.1%)	9 (5.0%)	34 (64.2%)	65 (65.0%)	9 (56.2%)
T + P + CT (*n*, %)	68 (47.2%)	1 (0.06%)	10 (18.9%)	22 (22.0%)	3 (18.8%)
Number of cycles 2019					
*n* (%)	1021 (50.2%)	1143 (49.5%)	109 (24.5%)	336 (39.8%)	41 (29.7%)
Mean (SD)	7.1 (6.4)	6.3 (5.9)	2.1 (3.7)	3.36 (5.0)	2.6 (4.8)
Range	1–24	1–19	1–14	1–18	1–14
Number of cycles 2020					
*n* (%)	1014 (49.8%)	1164 (50.5%)	336 (75.5%)	508 (60.2%)	97 (70.3%)
Mean (SD)	7.0 (6.6)	6.4 (6.3)	6.3 (4.6)	5.1 (4.7)	6.1 (4.9)
Range	1–19	1–19	1–17	1–18	1–17
Prior trastuzumab-based therapy					
Yes (*n*, %)	62 (43.4%)	130 (72.2%)	36 (69.2%)	33 (33.0%)	10 (62.5%)
No (*n*, %)	81 (56.6%)	50 (27.8%)	16 (30.8%)	67 (67.0%)	6 (37.5%)
Patients experiencing ADRs					
Yes (*n*, %)	18 (12.5%)	12 (6.7%)	6 (11.3%)	12 (12.0%)	5 (31.2%)
No (*n*, %)	126 (87.5%)	168 (93.3%)	47 (88.7%)	88 (88.0%)	11 (68.8%)

Abbreviations: IV—intravenous; SC—subcutaneous; T—trastuzumab; T + P—trastuzumab plus pertuzumab; T + CT—trastuzumab plus chemotherapy; T + P + CT—trastuzumab plus pertuzumab plus chemotherapy; ADRs—adverse drug reactions.

**Table 2:** Characteristics of switch from originator versus biosimilar and vice versa.

Switch from Originator to Biosimilar (*n*, %)	
Originator vs. Biosimilar (*n*, %)	34 (31.1%)
Biosimilar vs. Originator IV (*n*, %)	6 (5.4%)
Biosimilar vs. Originator SC (*n*, %)	55 (50.0%)
Biosimilar vs. Biosimilar (*n*, %)	15 (13.6%)

Abbreviations: IV—intravenous; SC—subcutaneous.

**Table 3:** Adverse drug reactions (ADRs) reported for each originator and biosimilar medicine.

	Herceptin IV (*n*, %)	Herceptin SC (*n*, %)	Herzuma (*n*, %)	Kanjinti (*n*, %)	Ontruzant (*n*, %)
	34 (41.0%)	16 (19.3%)	10 (12.0%)	15 (18.1%)	8 (9.6%)
Setting					
Neoadjuvant (*n*, %)	4 (4.8%)	3 (3.6%)	0	2 (2.4%)	2 (2.4%)
Adjuvant (*n*, %)	7 (8.4%)	5 (6.0%)	2 (2.4%)	7 (8.4%)	2 (2.4%)
Recurrent/Metastatic (*n*, %)	23 (27.7%)	8 (9.6%)	8 (9.6%)	6 (7.2%)	4 (4.8%)
Regimen					
T (*n*, %)	0	13 (15.7%)	3 (3.6%)	0	1 (1.2%)
T + P (*n*, %)	1 (1.2%)	0	0	1 (1.2%)	0
T + CT (*n*, %)	7 (8.4%)	3 (3.6%)	2 (2.4%)	11 (13.3%)	7 (8.4%)
T + P + CT (*n*, %)	26 (31.3%)	0	5 (6.0%)	3 (3.6%)	0
Severity					
Serious	4 (4.8%)	3 (3.6%)	4 (4.8%)	6 (7.2%)	5 (6.0%)
Nonserious	30 (36.1%)	13 (15.6%)	6 (7.2%)	9 (10.8%)	3 (3.6%)
ADRs description					
				
General disorders and administration site conditions					
Pyrexia, *n* (%)	1 (1.2%)	0	0	0	0
General discomfort, *n* (%)	1 (1.2%)	0	1 (1.2%)	0	0
Asthenia, *n* (%)	1 (1.2%)	0	0	1 (1.2%)	0
Edema limbs, *n* (%)	1 (1.2%)	1 (1.2%)	0	0	0
Chills, *n* (%)	1 (1.2%)	0	0	0	0
Gastrointestinal disorders					
Gastrointestinal pain, *n* (%)	0	0	0	0	1 (1.2%)
Diarrhea, *n* (%)	4 (4.8%)	0	1 (1.2%)	0	1 (1.2%)
Constipation, *n* (%)	1 (1.2%)	0	0	0	0
Gastroesophageal reflux, *n* (%)	0	1 (1.2%)	0	0	0
Erythema, *n* (%)	1 (1.2%)	2 (2.4%)	0	1 (1.2%)	0
Rash maculo-papular, *n* (%)	2 (2.4%)	0	1 (1.2%)	0	0
Pruritus, *n* (%)	1 (1.2%)	0	1 (1.2%)	1 (1.2%)	0
Nail changes, *n* (%)	1 (1.2%)	0	0	0	0
Skin hyperpigmentation, *n* (%)	1 (1.2%)	0	0	0	0
Infections and infestations					
Skin infection, *n* (%)	1 (1.2%)	1 (1.2%)	0	1 (1.2%)	0
Hepatic infection, *n* (%)	0	1 (1.2%)	0	0	0
Blood and lymphatic system disorders					
Anemia, *n* (%)	7 (8.4%)	1 (1.2%)	1 (1.2%)	3 (3.6%)	2 (2.4%)
Leukopenia, *n* (%)	1 (1.2%)	1 (1.2%)	0	0	0
Neutropenia, *n* (%)	0	0	1 (1.2%)	1 (1.2%)	1 (1.2%)
Thrombocytopenia, *n* (%)	0	1 (1.2%)	0	2 (2.4%)	0
Investigations					
GGT increased, *n* (%)	1 (1.2%)	0	0	0	0
Lipase increased, *n* (%)	0	1 (1.2%)	0	0	0
ALT/AST increased, *n* (%)	5 (6.0%)	0	0	2 (2.4%)	1 (1.2%)
Cardiac Disorders					
Sinus bradycardia, *n* (%)	0	1 (1.2%)	0	0	0
Sinus tachycardia, *n* (%)	1 (1.2%)	0	0	1 (1.2%)	0
Left ventricular systolic dysfunction, *n* (%)	0	0	0	1 (1.2%)	0
Atrioventricular block first degree, *n* (%)	1 (1.2%)	0	0	0	0
Other cardiac disorders, *n* (%)	0	0	1 (1.2%)	0	0
Vascular Disorders					
Hypertension, *n* (%)	1 (1.2%)	0	0	0	0
Respiratory, thoracic and mediastinal disorders					
Dyspnea, *n* (%)	0	0	1 (1.2%)	0	0
Musculoskeletal and connective tissue disorders					
Muscle weakness lower limb, *n* (%)	0	1 (1.2%)	0	0	1 (1.2%)
Metabolism and nutrition disorders					
Hyperuricemia, *n* (%)	0	2 (2.4%)	0	0	0
Ear and labyrinth disorders					
Middle ear inflammation, *n* (%)	0	1 (1.2%)	0	0	0
Nervous system disorders					
Peripheral sensory neuropathy, *n* (%)	2 (2.4%)	1 (1.2%)	2 (2.4%)	1 (1.2%)	1 (1.2%)
Blurred vision, *n* (%)	0	0	0	1 (1.2%)	0

Percentages refer to total number of ADRs registered (*n* = 83). Abbreviations: IV—intravenous; SC—subcutaneous; T—trastuzumab; T + P—trastuzumab plus pertuzumab; T + CT—trastuzumab plus chemotherapy; T + P + CT—trastuzumab plus pertuzumab plus chemotherapy; ADRs—adverse drug reactions.

## Data Availability

The data presented in this study are available on request from the corresponding author.

## Abbreviations

ADRs	Adverse drug reactions
BC	Breast cancer
EMA	European Medicine Agency
FDA	Food and Drug Administration
HER2	Human epidermal growth factor receptor-2
IV	Intravenous
MedDRA	Medical Dictionary for Regulatory Activities
SC	Subcutaneous
WHO	World Health Organization
